# A Novel Embedded Feature Selection and Dimensionality Reduction Method for an SVM Type Classifier to Predict Periventricular Leukomalacia (PVL) in Neonates

**DOI:** 10.3390/app112311156

**Published:** 2021-11-24

**Authors:** Dieter Bender, Daniel J. Licht, C. Nataraj

**Affiliations:** 1 Villanova Center for Analytics of Dynamic Systems, Villanova University, 800 Lancaster Ave, Villanova, PA 19085, USA; 2 June and Steve Wolfson Laboratory for Clinical and Biomedical Optics, Children’s Hospital of Philadelphia, 324 S 34th St, Philadelphia, PA 19104, USA

**Keywords:** periventricular leukomalacia, active learning, interactive machine learning, support vector machine, feature selection, dimensionality reduction, congenital heart disease, pediatric

## Abstract

This paper is concerned with the prediction of the occurrence of periventricular leukomalacia (PVL) in neonates after heart surgery. Our prior work shows that the Support Vector Machine (SVM) classifier can be a powerful tool in predicting clinical outcomes of such complicated and uncommon diseases, even when the number of data samples is low. In the presented work, we first illustrate and discuss the shortcomings of the traditional automatic machine learning (aML) approach. Consequently, we describe our methodology for addressing these shortcomings, while utilizing the designed interactive ML (iML) algorithm. Finally, we conclude with a discussion of the developed method and the results obtained. In sum, by adding an additional (Genetic Algorithm) optimization step in the SVM learning framework, we were able to (a) reduce the dimensionality of an SVM model from 248 to 53 features, (b) increase generalization that was confirmed by a 100% accuracy assessed on an unseen testing set, and (c) improve the overall SVM model’s performance from 65% to 100% testing accuracy, utilizing the proposed iML method.

## 1. Introduction

Artificial Intelligence (AI) and Machine Learning (ML) are the fastest growing fields in computer science, and are expediting progress in many other research fields [1,2]. With ever-growing computational power and quantities of data, AI has made significant advancements in numerous areas, including business analytics, speech recognition, system diagnostics/prognostics, and autonomous driving [2]. In particular, the introduction of AI and ML into clinical research has allowed the healthcare sector to make advances in the decision-making process through better detection and prediction of diseases in patients at an early stage ([2] pp. 289–302, [3,4]). However, despite the excellent solutions offered by the automatic ML (aML) [5] approach, its learning process has become increasingly complex and opaque, limiting its applicability in medical research [1,2].

In numerous medical fields, current research is confronted with limited data sets, attributed primarily to the rarity of events and the cost of data collection. As a result, the development of intelligent patient-monitoring and disease-predicting techniques is hampered by the generalization of the aML classifiers [6,7]. Furthermore, many medical problems are characterized by poorly understood causality of events [8]. For example, predicting rare diseases relies on knowledge-driven techniques to pinpoint what is important in the data and incorporate this into the ML model. Against this backdrop, there is an urgent need to enhance the transparency and generalization of ML algorithms.

In particular, ML models play a crucial role in identifying singular disorders or diseases in infants. Our previous study applied ML to predict the occurrence of a rare brain injury known as periventricular leukomalacia (PVL) in neonates following congenital heart surgery [9]. Hypoplastic left heart syndrome (HLHS) and transposition of the great arteries (TGA) are two cardiac disorders that usually need surgical intervention in infancy and are linked with increased risks of brain damage [8]. The occurrence of PVL after cardiac surgery has been linked to physiological disorders such as hypoxemia, reduced cerebral blood flow, and low arterial carbon dioxide levels [9,10].

**Figure 1.**
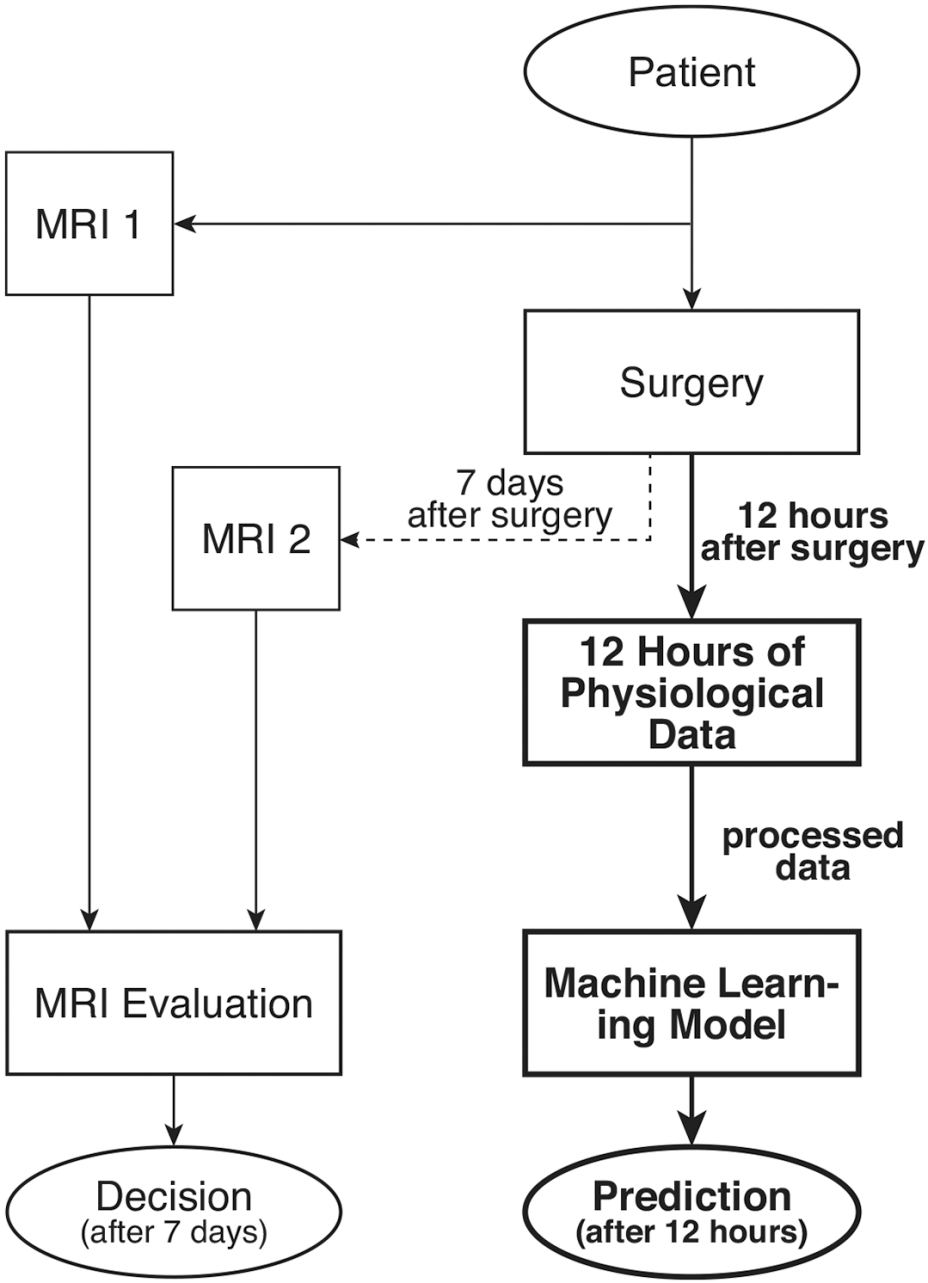
Current decision method after 7 days and proposed predictive method (bold) after 12 h.

Although the pathology of PVL is somewhat understood, predicting its occurrence in neonates has thus far remained a challenge since the origins of this rare condition remain to be recognized [9,11]. Currently, clinicians rely on magnetic resonance imaging (MRI) to diagnose a neonate with PVL. Usually, one MRI is carried out on a patient just before the surgery and one about a week after [12,13]. By comparing the white brain tissue near the ventricles captured in the MRIs, a clinician can infer if PVL has occurred. Figure 1 illustrates the current approach and compares it with the novel predictive ML method used in our previous study [14].

While the predictive model introduced in our previous studies [14-18] provided satisfying classification results, it did not explain them. That is, the algorithm had no transparency, providing no indication of which portion of the data was relevant to the performance of the ML algorithm. Hence, in this study, we incorporate a derived step from Active Learning (AL), to determine which part of the data was decisive in making the prediction and, further, to explore the meaning of these data from a physiological perspective [19]. In summary, with a limited amount of data samples, the goal of this study is to develop an interactive ML method that lowers the dimensionality of the final disease-predicting model [20] while increasing generalization and overall performance for future unknown data.

For this purpose, we first illustrate and explain the shortcomings of the traditional aML approach. Subsequently, we describe our methodology addressing these shortcomings while utilizing interactive ML (iML) [5]. Finally, we conclude with a discussion of the developed method and the results obtained.

## 2. Methods

This section illustrates the methods that were used to address the challenges presented in the introduction. First, it presents the raw data and provides context for understanding the overall necessity of the proposed methods. The second subsection briefly presents the ML data in its feature space, and the third discusses the need for feature selection. The benchmark and the developed methods are presented in the following subsections, where the latter depicts the limitations of the benchmark methodology and presents the established methods to address those.

### 2.1. Raw Data

The raw physiological data was collected following a pre-specified protocol at Children’s Hospital of Philadelphia (CHOP) and approved for this retrospective study. Patients (*N* = 56), term neonates (gestational age [GA] > 37 weeks) with congenital heart disease (CHD) who underwent cardiac surgery during the first 30 days of life, were monitored postoperatively in a cardiac intensive care unit (CICU). During the post-operative monitoring, physiological data, including heart rate (HR), mean arterial blood pressure (MAP), right atrial pressure (RAP), and oxygen saturation (SpO2), was recorded for 12 h directly following the surgery. These four signals monitoring the heart’s health are believed to be carrying significant indicators for PVL occurrence, particularly since PVL injury in neonates is due to, but not limited to, the effects of various interventions such as cardiac surgery required to treat children with complex congenital heart diseases, such as HLHS and TGA [21]. PVL injury in each neonate was inferred from the MRI comparison by a trained physician (Figure 1), where PVL positive (*p* = 32) denoted as patients with evidence of intracranial hemorrhage in size larger than 100 mm^3^ and PVL negative (*n* = 24) denoted such patients with intracranial hemorrhage smaller than 10 mm^3^. Figure 1 illustrates the data collection’s temporal path and contrasts the shortcomings of the current PVL diagnosis and the developed predictive model’s superiority.

### 2.2. Machine Learning Data

For each data sample (neonate), the characteristics of the four physiological measurements were extracted using wavelet transform and assigned as features. The resulting ML data set consists of 56 samples, each with a 248-dimensional input vector of features. The output vector, PVL occurrence (PVL positive = 1, PVL negative = 0) in each neonate, was inferred from the MRI comparison by a trained physician (Figure 1). Considering this study’s objective, the details of the feature extraction process are excluded from this paper but can be found in [14].

### 2.3. Feature Selection

A general phenomenon, overfitting, occurs with all types of learning algorithms and is often the effect of large dimensionality in the feature space of the generated predictive model [22,23]. This section illustrates two feature subset selection methods to reduce the dimensionality of the model, with their relation to the performance and generalization of the classification model. First, the benchmark method used in earlier work [14] is described to highlight its shortcomings and the differences in the developed iML algorithm, the concepts of which are described in the latter part of this section.

#### 2.3.1. Filter Method

To optimize the ML model published by Jalali and colleagues [14,15], the team used a filter approach as a benchmark method to select the best possible feature subset. This method aims to establish a relevance metric based on selected correlation (or dependency) criteria between individual features and the output, ranking them from weak to strong [23-25]. In this case, the relevance metric was established based on the computed mutual information (MI) value for each feature [26-28]. Based on a user-specified relevance threshold of the established MI-metric, the best possible feature subset was selected and passed on to the ML algorithm.

**Figure 2.**
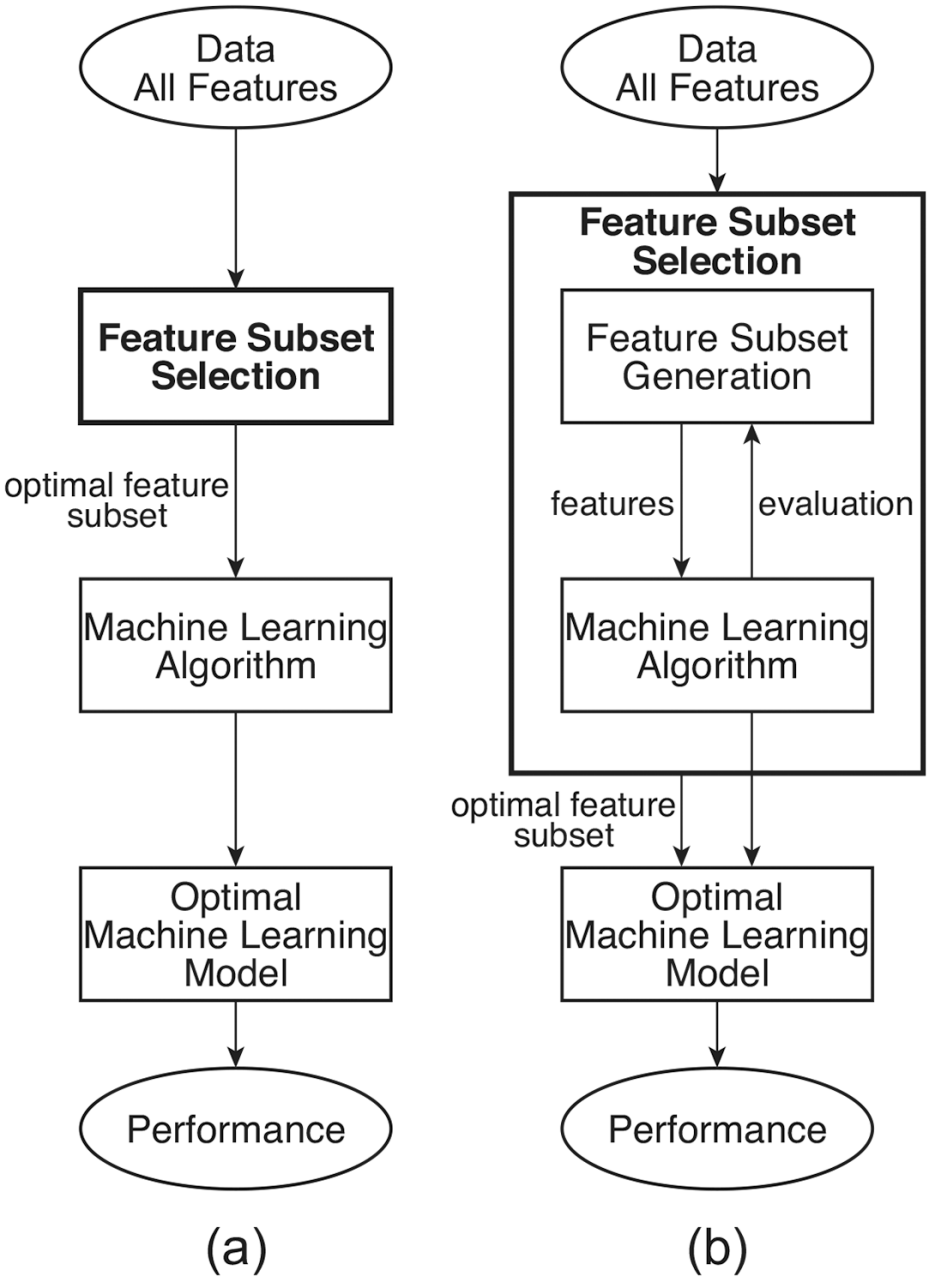
Dimensionality reduction and most relevant feature subset selection: (**a**) Filter method and (**b**) Wrapper method.

Generally, as shown in Figure 2a, the user-specified threshold value is adjusted until the best possible ML training performance is achieved. It should be noted that the filter step is independent of the learning algorithm since it is established before the ML training start; thus, as shown in the results section, the same rank of features was used with four different types of ML algorithms: (a) Simple Tree with optimal pruning of 13 parents; (b) Radial Basis Function kernel Support Vector Machine (RBF SVM) with a standard γ calculated from the number of features used; (c) Linear SVM with automatically optimized hyper-parameters; (d) *k*-Nearest Neighbor (*k*NN) with euclidean distance, no distance weighting and 4 nearest neighbors.

#### 2.3.2. Wrapper Method

As illustrated in the first part of the results section and explained in the discussion section, the previously used benchmark filter method [14,15] suffers from several crucial drawbacks. To address these drawbacks, the invented feature selection algorithm presented in this study builds on the idea of the wrapper method, shown in Figure 2b. The wrapper method structure is as follows: the feature subset selection algorithm searches for the “best possible” subset using a selected ML algorithm itself as part of the feature subset evaluating function [23,25]. In this structure, the selected ML algorithm is trained on the dataset, usually partitioned into internal training and validation sets, with different subsets of features removed from or added to the data. The feature subset with the highest evaluation is chosen as the final set on which to train the final ML model. In this arrangement, the ML algorithm in the wrapper structure is often considered to be a black box and is consequently evaluated during the training stage, either only on various validation sets or ultimately on an independent testing set that was not used during the training process. Although this approach gives better performance than the filter method, it is computationally expensive and, more importantly, prone to overfitting.

In the established algorithm, we resolve the over-fitting of the wrapper method, which originates primarily from the bias of the training data, with two major advances. First, the black-box framework is discarded, and an optimization algorithm is embedded into the learning structure of the ML algorithm, rendering the feature selection process part of the ML model development. Second, the fitness (cost) function of the optimization algorithm is built such that the optimized model is independent of the training results. As the feature domain is discontinuous, the specified optimization problem requires a guided random search technique. As a result, a Genetic Algorithm (GA) was chosen as an optimization algorithm.

By natural design, the GA explores the population of points in the given domain using probabilistic and global heuristic search [29]. This makes optimization resilient to local minima/maxima, enabling investigation of the importance of features and their combination across the entire feature domain [23]. Furthermore, GA performs well when the fitness function, which is an objective function used to direct genetic programming towards an optimal design solution, is complex and defined as a mixed (discrete and continuous) multi-objective problem, as it is in this optimization process, described by the fitness function FF in Equations (1)-(4).

(1) $$\underset{FF_{1},FF_{2},FF_{3}}{\text{argmin}}FF={FF}_{1}+{FF}_{2}+{FF}_{3}$$

(2) $$\underset{ACC_{train}}{\text{argmin}}{FF}_{1}=\frac{1}{{ACC}_{train}}$$

(3) $$\underset{m}{\text{argmin}}{FF}_{2}=a\frac{1}{m}$$

(4) $$\underset{\left\|w_{m}\right\|}{\text{argmin}}{FF}_{3}=b\frac{1}{\left\|w_{m}\right\|},$$

where m is the subset’s number of features, a and b are weight parameters, and $\left\|w_{m}\right\|$ is the width of the separating plane in $m^{th}$ dimension.

The fitness function FF was created with the objective to minimize the subset of features while striving to achieve three goals. First, ${FF}_{1}$ ensures the model’s accuracy with respect to the training set. Second, the ${FF}_{2}$ term ensures that the total number of features was minimized. Ultimately, the ${FF}_{3}$ term has been established to direct the ML model to a state with the greatest potential for generalization without relying on the training set as feedback.

**Figure 3.**
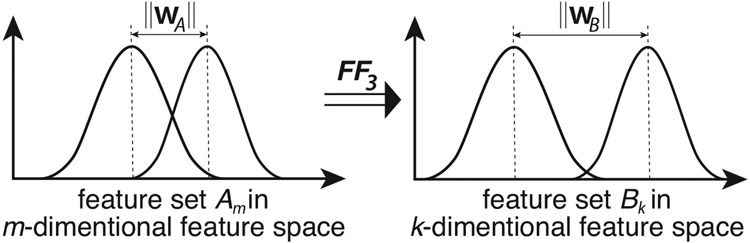
Optimization function ${FF}_{3}$ in terms of set distribution, where the set $B_{k}\;\in \;A_{m}$, k≤m and m is the number of all features.

The idea behind the design of the ${FF}_{3}$ function is based on the geometric concept in the SVM structure and the notion of separating distance between two sets of classes [7,22,23,30-32]. It was hypothesized that if both classes A, B are of a normal distribution, the greater the dividing margin between A, B in an m-dimensional space, the lower the overlapping likelihood (A∩B) between the two classes in that space. Based on this fundamental concept, as illustrated in Figure 3, this remains statistically valid for any normal data set and can be used as a dimensionality reduction or feature optimization method for any SVM type learning algorithm.

Without detailed derivation, mathematically the SVM is expressed as a dual optimization problem by:

(5) $$\underset{\alpha }{\text{argmax}}\sum_{i}\alpha_{i}-\frac{1}{2}\sum_{i}\sum_{j}\alpha_{i}\alpha_{j}y_{i}y_{j}(x_{i}\cdot x_{j}),$$

subject to the constraints $\alpha_{i}\geq 0$ and $\sum_{i}\alpha_{i}y_{i}=0$, and where x is the input vector, y is the output vector and the vector α is the Lagrange multipliers. Thus, as stated by the dual form of the SVM optimization problem (5), searching for the maximum-margin decision boundary is analogous to searching for the support vectors (SVs) $x_{i}$, for which $\alpha_{i}\neq 0$ and the entire decision boundary can be described as follows:

(6) $$w=\sum_{i}\alpha_{i}y_{i}x_{i}$$

Thereby, the aim of ${FF}_{3}$ in Equation (4) is to find a subset of features with maximum decision boundary margin, expressed by the euclidian distance measure $\left(2\text{-}norm)\;\|w_{m}\right\|$ of $w_{m}=\sum_{i}\alpha_{i}y_{i}x_{i}$, with m being the number of features or the reduced dimensionality of the learning model.

The entire optimization process is presented as pseudocode in Algorithm 1, covering all of the key actions. In order to monitor the performance of the designed optimization algorithm at each iteration, the best SVM model was evaluated on an unseen testing set with the feature vector and the dimensionality determined by the model from each optimization epoch.

**Table:** 

Algorithm 1 Embedded Feature Subset Optimization Algorithm
$$\begin{array}{cc} \; & \;\mathrm{function}\;\text{GENETIC-ALGORITHM}(population,\text{FITNESS-FUNCTION})\\ \; & \;\;\mathrm{inputs}:population,\text{set of}\;c\;\text{random feature subsets}\;\;\;\;\;\;\;\;\;\;\;\;\;\;\;\;\;\;\;\;\;\;\;\;\;\;\;\;\;\;\;\;\;\;\;\;\;\;\;\;⋄\;c:\text{number of chromosomes}\\ \; & \;\\ \; & \;\;\;\;\mathrm{repeat}\\ \; & \;\;\;\;\;new\_population\leftarrow \text{empty set}\\ \; & \;\;\;\;\;\;\;\;\mathrm{for}\;i=1\;\mathrm{to}\;\text{SIZE}(population)\;\mathrm{do}\\ \; & \;\;\;\;\;\;\;\;\;\;\;x\leftarrow \text{RANDOM-SELECTION}(population,\text{FITNESS-FUNCTION})\;⋄\;x\;\text{is selected random w.r.t.}\;fitness\text{-}score\;\text{as its}\\ \; & \;\text{probability}\\ \; & \;\;\;\;\;\;\;\;\;\;\;y\leftarrow \text{RANDOM-SELECTION}(population,\text{FITNESS-FUNCTION})\;⋄\;y\;\text{is selected random w.r.t.}\;fitness\text{-}score\;\text{as its}\\ \; & \;\text{probability}\\ \; & \;\;\;\;\;\;\;\;\;\;\;child\leftarrow \text{REPRODUCE}(x,y)\\ \; & \;\;\;\;\;\;\;\;\;\;\;\mathrm{if}\;\text{small random probability}\;\mathrm{then}\;child\;\leftarrow \;\text{MUTATE}(child)\;\;\;\;\;\;\;\;\;\;\;\;\;⋄\;\text{this probability is defined by a selected}\\ \; & \;mutation\text{-}rate\\ \; & \;\;\;\;\;\;\;\;\;\;\;\text{add}\;child\;\text{to}\;new\_population\\ \; & \;\;\;\;\;\;\;\;population\leftarrow new\_population\\ \; & \;\;\;\;\mathrm{until}\;\text{solution is found that satisfies minimum criteria},\;\text{or enough}\;generation\;\text{have elapsed}\\ \; & \;\mathrm{return}\;\text{the best set in}\;population,\text{faccording to}\;\text{FITNESS-FUNCTION}\;\;\;\;\;\;\;\;\;\;\;\;\;\;\;\;⋄\;\text{best feature subset, according to the}\\ \; & \;\text{FITNESS-FUNCTION}\\ \; & \;\underline{\;\;\;\;\;\;\;\;\;\;\;\;\;\;\;\;\;\;\;\;\;\;\;\;\;\;\;\;\;\;\;\;\;\;\;\;\;\;\;\;\;\;\;\;\;\;\;\;\;\;\;\;\;\;\;\;\;\;\;\;\;\;\;\;\;\;\;\;\;\;\;\;\;\;\;\;\;\;\;\;\;\;\;\;\;\;\;\;\;\;\;\;\;\;\;\;\;\;\;\;\;\;\;\;\;\;\;\;\;\;\;\;}\\ \; & \;\mathrm{function}\;\text{REPRODUCE}(x,y)\\ \; & \;\;\mathrm{inputs}:x,y,\;\text{two chromosomes from the population}\;\;\;\;\;\;\;\;\;\;\;\;\;\;\;\;\;\;\;\;\;\;\;\;\;\;\;⋄\;\text{evaluated by the FITNESS-FUNCTION}\\ \; & \;\\ \; & \;\;\;\;n\leftarrow \text{LENGTH}(x);\\ \; & \;\;\;\;l\leftarrow \text{number from}\;1\;\text{to}\;n\;\;\;\;\;\;\;\;\;\;\;\;\;\;\;\;\;\;\;\;\;\;\;\;\;\;\;\;\;\;\;\;\;\;\;\;\;\;\;\;\;\;\;\;\;\;\;\;\;\;⋄\;l\;\text{is defined by a selected}\;crossover\text{-}rate\\ \; & \;\;\;\;child\leftarrow \text{APPEND}(\text{SUBSTRING}(x,1,l),\text{SUBSTRING}(y,l+1,n))\;\;\;\;\;\;\;\;\;\;\;\;\;\;\;\;\;\;\;\;\;\;\;\;\;\;\;\;\;\;\;⋄\;\text{new chromosome}\\ \; & \;\mathrm{return}\;child\\ \; & \;\underline{\;\;\;\;\;\;\;\;\;\;\;\;\;\;\;\;\;\;\;\;\;\;\;\;\;\;\;\;\;\;\;\;\;\;\;\;\;\;\;\;\;\;\;\;\;\;\;\;\;\;\;\;\;\;\;\;\;\;\;\;\;\;\;\;\;\;\;\;\;\;\;\;\;\;\;\;\;\;\;\;\;\;\;\;\;\;\;\;\;\;\;\;\;\;\;\;\;\;\;\;\;\;\;\;\;\;\;\;\;\;\;\;}\\ \; & \;\mathrm{function}\;\text{FITNESS-FUNCTION}(population)\;\;\;\;\;\;\;\;\;\;\;\;\;\;\;\;\;\;\;\;\;\;\;\;\;\;\;\;\;\;\;\;\;\;\;\;\;\;\;\;\;\;\;\;\;\;\;\;\;\;\;\;\;\;\;\;\;\;\;\;\;⋄\;\text{user defined}\\ \; & \;\;\mathrm{inputs}:population,\;\text{a set of}\;c\;\text{random feature subsets}\;\;\;\;\;\;\;\;\;\;\;\;\;\;\;\;\;\;\;\;\;\;\;\;\;\;\;\;\;\;\;\;\;\;\;\;\;\;⋄\;c:\text{number of chromosomes}\\ \; & \;\\ \; & \;\;\;\;\mathrm{for}\;j=1\;\mathrm{to}\;\text{SIZE}(population)\;\mathrm{do}\\ \; & \;\;\;\;\;\;\;m\leftarrow \text{SIZEe}(population(j))\;\;\;\;\;\;\;\;\;\;\;\;\;\;\;\;\;\;\;\;\;\;\;\;\;\;\;\;\;\;\;\;\;\;\;\;\;\;\;\;⋄\;m\;\text{is the dimensionality of}\;j^{th}\;\text{chromosome}\\ \; & \;\;\;\;\;\;\;SVM\text{-}model\leftarrow \text{TRAIN-SVM}(\text{Training-Samples}(population(j))\\ \; & \;\;\;\;\;\;\;{ACC}_{train}\leftarrow \text{accuracy of the}\;SVM\text{-}model\;\text{on the Training-Samples}\\ \; & \;\;\;\;\;\;\;w_{m}\leftarrow \text{DECISION-BOUNDARY}(SVM\text{-}model)\\ \; & \;\;\;\;\;\;\;\|w_{m}\leftarrow \text{MAGNITUDE}(w_{m})\;\;\;\;\;\;\;\;\;\;\;\;\;\;\;\;\;\;\;\;\;\;\;\;\;\;\;\;\;⋄\;\text{the}\;2\text{-}norm\;\text{of}\;\|w_{m}\|=\text{decision boundary margin}\\ \; & \;\;\;\;\;\;\;{FF}_{1}\leftarrow \frac{1}{{ACC}_{train}}\\ \; & \;\;\;\;\;\;\;{FF}_{2}\leftarrow a\frac{1}{m}\;\;\;\;\;\;\;\;\;\;\;\;\;\;\;\;\;\;\;\;\;\;\;\;\;\;\;\;\;\;\;\;\;\;\;\;\;\;\;\;\;\;\;\;\;\;\;\;\;\;\;\;⋄\;a\;\text{is a user defined weight parameter of}\;{FF}_{2}\\ \; & \;\;\;\;\;\;\;{FF}_{3}\leftarrow b\frac{1}{\left\|w_{m}\right\|}\;\;\;\;\;\;\;\;\;\;\;\;\;\;\;\;\;\;\;\;\;\;\;\;\;\;\;\;\;\;\;\;\;\;\;\;\;\;\;\;\;\;\;\;\;\;\;\;⋄\;b\;\text{is a user defined weight parameter of}\;{FF}_{3}\\ \; & \;\;\;\;\;\;\;\mathrm{FF}(j)\leftarrow {FF}_{1}+{FF}_{2}+{FF}_{3}\;\;\;\;\;\;\;\;\;\;\;\;\;\;\;\;\;\;\;\;\;\;\;\;\;\;\;\;\;\;\;\;\;\;\;\;\;\;\;\;⋄\;fitness\text{-}score\;\text{of the}\;j^{th}\;\text{chromosome}\\ \; & \;\;\;\;fitness\text{-}score\leftarrow \mathrm{FF}\;\;\;\;\;\;\;\;\;\;\;\;\;\;\;\;\;\;\;\;\;\;\;\;\;\;\;\;\;\;\;\;\;\;\;\;\;\;\;\;\;\;\;⋄\;\text{fitness-scores for all chromosomes in the}\;population\\ \; & \;\mathrm{return}\;fitness\text{-}score \end{array}$$

## 3. Results

**Figure 4.**
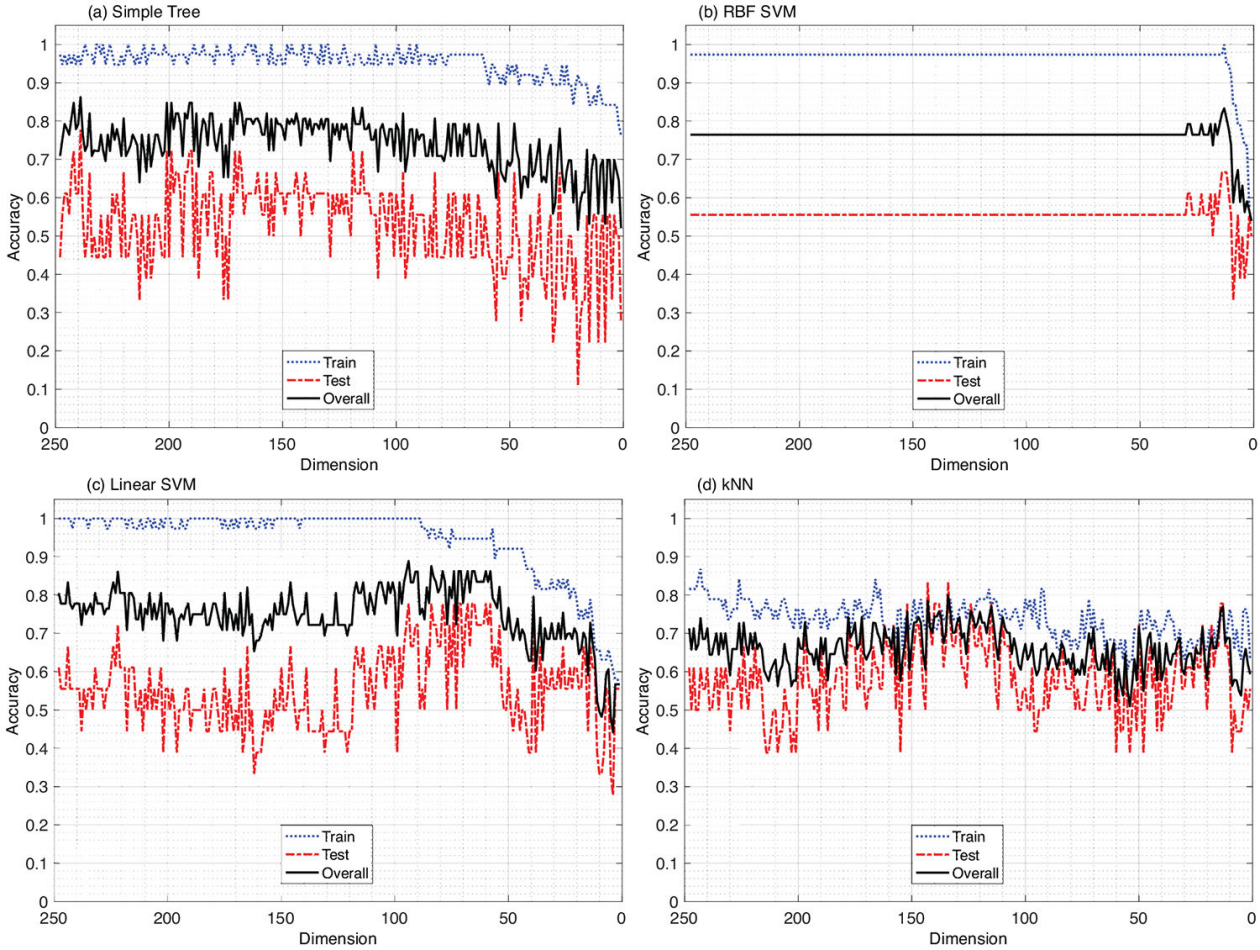
Filter method (benchmark): Four machine learning algorithms evaluated with respect to sequential dimensionality reduction using the mutual information relevance metric. (**a**) Simple Tree, (**b**) Radial Basis Function kernel Support Vector Machine, (**c**) Linear Support Vector Machine, (**d**) *k*-Nearest Neighbor. Data: *N* = *Overall* = 56 (*p* = 32, *n* = 24), *R* = *Train* = 38 (*p* = 23, *n* = 15), *E* = *Test* = 18 (*p* = 9, *n* = 9).

When the filter method was used to minimize the dimensionality of the ML model, where the defined MI metric was used to sequentially delete the least significant features, the results did not always translate into the model’s best possible performance on an unknown testing set. Figure 4 depicts the relationship between the model’s training, testing, and overall accuracy as the dimensionality is reduced. This relationship is illustrated using four different types of machine learning models: (a) Simple Tree, (b) RBF SVM, (c) Linear SVM, and (d) kNN. The wrapper method is applied as a consequence of these benchmark results, which are covered in depth in the discussion portion of this paper.

**Figure 5.**
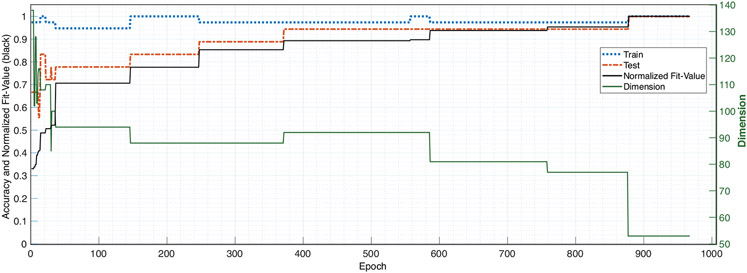
Feature Optimization Results (left y-scale) of the Embedded Genetic Algorithm Wrapper Method and the Dimension Reduction (right y-scale). Data: *N* = 56 (*p* = 32, *n* = 24), *R* = 38 (*p* = 23, *n* = 15), *E* = 18 (*p* = 9, *n* = 9).

The findings indicate that the overall performance of the final ML model obtained the best possible outcome by utilizing the embedded wrapper approach. As is evident from Figure 5, we were able to create a feature set optimization algorithm that guides the formation of the ML model to the state with the highest potential for generalization. Figure 5 shows that, the dimensionality is reduced to 53 features, there is a strong relationship between the optimization fit-value (fitness-function) and the individual outputs of the ML model (training, testing, and overall accuracy). The final state of the GA optimization achieves 100% overall accuracy after 893 epochs.

The importance of these results is examined and evaluated thoroughly in the following section.

## 4. Discussion

The results of this investigation clearly highlight the weaknesses of the (benchmark) filter method. Its main drawback is highlighted in Figure 4, where no clear association between the relevance of the removed features and the model’s performance trend can be identified. In Figure 4a,c, no discernible impact of dimensionality reduction on the model’s accuracy can be seen until it is reduced to nearly 80 features, and thereafter, the model’s overall performance begins to decline. Figure 4b illustrates the same pattern, except in a more dramatic manner, where the dimensionality of the model has little effect on the model’s performance until it is reduced to fewer than 24 features.

One might claim that the Linear SVM at about 70 features and the RBF SVM at 19 features achieve their optimal performance; however, there is no perceptible information-gain-trend before or after these peak outcomes that can be identified with the filter method’s dimensionality reduction. Figure 4d shows some of the agreements between the dimensionality of the model and its performance, with the overall accuracy (red) of the model showing an upward rise, achieving its best performance with about 160 features. However, as with the other three ML models, while the features are excluded sequentially from the data, it is unclear how to establish the threshold point for rankings, which must be defined by the user in order to include only the necessary features and exclude the redundant ones. As a consequence, the only way the interaction between the filter method and the ML models can be measured is through the performance of the model at the training or the testing stage, which essentially results in a more data-biased classification model, providing no clear indication that the generalization of the model was improved through dimensionality reduction using the filter method.

Unlike the filter method, the ML model using the embedded wrapper approach and GA optimization, generated far more satisfying results. As shown in Figure 5, we were able to create a feature-set optimization algorithm that guides the development of the ML model to the best possible state, with a maximum accuracy of 100%. The fit-value (fitness function), represented by the solid black line in Figure 5, demonstrates a consistent relationship with the model’s training (dotted blue), testing (dotted red) accuracy, and decreased dimensionality, represented by a solid green line with its scale on the right side of the same plot. Consequently, given that the fitness function (1)-(4) of the optimization was entirely independent of the testing data and only partly reliant on the training data (2), it is clear that the maximum accuracy was achieved solely as a result of the ML model’s decreased dimensionality and increased generalization.

The high generalization and maximum precision are evidently attributable to the innovative use of the mathematical framework of the SVM (5), (6) on which ${FF}_{3}$ (4) was designed, something that can only be incorporated using the wrapper method. Furthermore, the strong results can be due to the combination of features investigated in the whole feature domain. In comparison to the filter method and certain wrapper methods, we were able to integrate the features’ dependencies on each other into the feature-selection cycle. This was accomplished thanks to the probabilistic and heuristic structure of the GA. The feature dependencies also became evident when the chosen number of chromosomes, one of the GA hyper parameters, was selected to be small (<300). In this scenario, the fitness function’s optimum fit-value would be obtained after just a few epochs (<100), because it would settle around local minima with a feature combination close to the starting one. Thus, the best GA optimization results were obtained for a large number of chromosomes, to account for feature dependencies across the entire feature domain, while avoiding local minima.

The best results of the embedded GA optimization wrapper were achieved with the GA hyper parameters as follows: *Number of Chromosomes* = 1200, *Maximum Number of Generations (Epochs)* = 1005, *Crossover Rate* = 0.8, *Mutation Rate* = 0.01. To attain the settling fit-value of 1 (100% accuracy), the developed algorithm took 38 h on a 64 bit MacBook Pro (MacOS 11.2.3), with a 3.1 GHz Quad-Core Intel Core-i7 processor and a 16 GB (2133 MHz LPDDR3) memory using MathWorks MATLAB R2019a (9.6.0.1072779).

While the algorithm was tested many times and yielded consistent results, we admit that the restricted computational power and resulting long optimization time hindered deeper analysis of our algorithm. A high performance computational cluster will be used in the future to investigate the developed feature set optimization algorithm with differently weighted fitness functions (Equations (3) and (4)), additional variations of the GA hyper parameters, and in conjunction with various meta-heuristic algorithms.

## 5. Conclusions

The importance of dimensionality reduction and generalization of a machine learning model has been explicitly demonstrated in this paper. As validated by the provided work, it is advantageous to keep the dimensionality and hence the number of features low, even more so when dealing with a limited sample set. When examining the trajectory of the GA optimization, a clear correlation between the dimensionality, generalization, and performance of the ML model was observed, suggesting that the assumptions utilized to design the objective function were accurate. Furthermore, the findings describe significant differences between the filter and wrapper feature selection methods and conclude that the wrapper approach is superior. This is a critical observation since it validates the combinatorial advantages of the features, which the filter approach overlooks. The embedded wrapper approach and favorable results from the GA optimization demonstrated once more the importance of feature dependencies among themselves. Using the developed iML algorithm, we were able to create a feature set optimization algorithm that guides the training of the SVM model to the state with the highest potential for generalization, while improving the classification accuracy on an unseen testing set from 65% to 100%. Most notably, we were able to identify some of the most significant prognostic features for PVL occurrence in neonates by reducing the dimensionality of the model from 248 to 53 features. Expanding on this concept, the researchers are now able to extend their work through a different approach, to better predict and explain this form of pediatric brain injury. Additionally, based on the reported findings, future research can focus on engineering stronger features to enhance the ML model’s performance further.
